# Activity Strength within Optic Flow-Sensitive Cortical Regions Is Associated with Visual Path Integration Accuracy in Aged Adults

**DOI:** 10.3390/brainsci11020245

**Published:** 2021-02-16

**Authors:** Lauren Zajac, Ronald Killiany

**Affiliations:** 1 Department of Anatomy & Neurobiology, Boston University School of Medicine, 72 East Concord Street (L 1004), Boston, MA 02118, USA; killiany@bu.edu; 2 Center for Biomedical Imaging, Boston University School of Medicine, 650 Albany Street, Boston, MA 02118, USA

**Keywords:** spatial navigation, fMRI, aging, path integration, optic flow

## Abstract

Spatial navigation is a cognitive skill fundamental to successful interaction with our environment, and aging is associated with weaknesses in this skill. Identifying mechanisms underlying individual differences in navigation ability in aged adults is important to understanding these age-related weaknesses. One understudied factor involved in spatial navigation is self-motion perception. Important to self-motion perception is optic flow–the global pattern of visual motion experienced while moving through our environment. A set of optic flow-sensitive (OF-sensitive) cortical regions was defined in a group of young (*n* = 29) and aged (*n* = 22) adults. Brain activity was measured in this set of OF-sensitive regions and control regions using functional magnetic resonance imaging while participants performed visual path integration (VPI) and turn counting (TC) tasks. Aged adults had stronger activity in RMT+ during both tasks compared to young adults. Stronger activity in the OF-sensitive regions LMT+ and RpVIP during VPI, not TC, was associated with greater VPI accuracy in aged adults. The activity strength in these two OF-sensitive regions measured during VPI explained 42% of the variance in VPI task performance in aged adults. The results of this study provide novel support for global motion processing as a mechanism underlying visual path integration in normal aging.

## 1. Introduction

Spatial navigation is a complex, multisensory, cognitive skill critical to successful interaction with our environment, and aging is associated with weaknesses in this skill [1]. Fundamentally, navigating space, or traveling from one place to another, requires understanding and updating our location within our environment as we move through it. A number of sensory and environmental cues, component processes, and types of spatial knowledge or spatial representations might be employed to navigate to a desired location; the specific cues, processes, and representations used depend on the context as well as the individual who is navigating [2,3,4]. One cue that has been relatively understudied in the context of understanding individual differences in environmental spatial abilities and the mechanisms underlying weakened spatial abilities in aged adults is optic flow. Optic flow is the coherent, radial motion pattern experienced when moving forward in a stable environment with one’s head and eyes facing forward [5]. This characteristic global motion pattern provides information about the direction and speed of our self-motion as we move through our environment.

Many cortical areas that respond more strongly to optic flow than to other types of motion patterns have been identified in humans using fMRI [6,7,8,9]. Despite the close association between the sensitivity of these regions to visual motion patterns important to self-motion perception and spatial navigation, studies focused on these regions in the literature on aging and spatial navigation are lacking. Many of these optic flow-sensitive (OF-sensitive) regions are potentially homologous to regions initially identified electrophysiologically in non-human primates [10,11,12]. Several of these regions have also been shown to contain neurons tuned to the direction of self-motion (i.e., heading direction) based on optic flow alone [12]. The preferential response to optic flow in these cortical areas implicates them as playing a role in self-motion perception, and ongoing research is aimed at identifying which of these regions play significant roles in self-motion perception [12,13,14].

Studies of OF-sensitive regions have identified a set of characteristics that can be used to determine the degree to which a region is likely associated with self-motion and heading perception, and therefore the degree to which it is likely associated with navigational ability. According to these criteria, regions containing neurons that (1) are sensitive to and/or selective for radial motion, (2) show tuning for heading direction, (3) exhibit compensation for eye movements in their heading tuning, and (4) show congruent responses to visual and vestibular self-motion cues all implicate a region as being involved in self-motion and heading perception. Two regions in the macaque brain that satisfy these criteria are the medial superior temporal (MST) area (specifically dorsal MST) and the ventral intraparietal (VIP) area [12,13]. Regions potentially homologous to MST and VIP have been identified in humans using fMRI, as well [15,16,17,18,19], and these particular regions or others in their vicinity have been shown to be sensitive to heading direction based on visual motion information in humans using fMRI [20,21,22,23]. Though there is strong evidence implicating MST and VIP in self-motion perception, they nevertheless function in the context of an OF-sensitive network, the other members of which (such as V6 or PIVC) may be important to their function and self-motion perception, as well [10,14,24].

Understanding whether and how strongly OF-sensitive regions are involved in self-motion perception is important in its own right, but it is also important to understanding factors that explain variation in spatial navigation abilities. Optic flow perception contributes to our ability to form representations of our environment and our movement within it [25,26,27,28,29,30]. Supporting this, alterations in aspects of optic flow perception have been associated with spatial navigation deficits that are common in Alzheimer’s disease (AD) [31,32,33,34]. Optic flow perception has been hypothesized to be one factor that may underlie weakened spatial navigation abilities, particularly path integration, in normal aging, as well [1,4]. This is in part due to studies that have demonstrated that aging is associated with less accurate heading estimations based on optic flow [35] and in part due to the link between optic flow perception and navigation in AD noted above. However, there is a paucity of fMRI studies that focus on these questions, particularly in the context of aging.

The small number of studies that have used fMRI to investigate the effects of aging on the neural systems supporting spatial navigation have focused less on real-time spatial orienting and more on spatial memory and the formation of spatial representations [36,37,38,39,40,41]. All of these studies required participants to form a mental representation of a set of locations in space or routes and tested the accuracy of these representations after a delay. Spatial or sequence memory were intentionally embedded in all of these studies, partially because at least one goal of all of these studies was to examine differences in activity in medial temporal lobe structures between young and aged adults during such tasks. This goal was similarly motivated by the aging literature, the rodent literature, and the navigation literature in young adults, all of which show connections between allocentric spatial representations, episodic memory, aging, and the medial temporal lobe. The results of these fMRI studies support age-related differences in the neural systems supporting spatial learning in aged and young adults with some less effective at supporting spatial learning tasks and others potentially equally as effective. In terms of the medial temporal lobe, they support weaker hippocampal [36,38,39] and parahippocampal [37] activity during spatial and route learning in some contexts, a shift in the environmental cues to which the hippocampus is sensitive in others [40], and instability in the entorhinal grid system during navigation to remembered locations [41]. These studies are informative in providing a foundation for our understanding of differences in the neural systems supporting spatial and route learning and memory in aged adults and aspects of these systems associated with the strength of these abilities. However, aside from a few exceptions, these studies do not examine or inform us in detail about the mechanisms associated with tracking one’s position and orientation while moving through space, which is fundamental to spatial navigation. Presumably, sensory and perceptual processes important to self-motion perception and real-time spatial orienting would be relevant and important to the formation, retrieval, and use of spatial representations. Yet, fMRI studies of aging and spatial navigation that focus on sensory processes are lacking despite evidence for a connection between optic flow perception and spatial navigation and the ability to identify and measure signal from cortical regions sensitive to optic flow and implicated in self-motion perception. The experiments in this study were designed to address this gap in the literature. We set out to test the hypothesis that activity strength in OF-sensitive cortical regions is associated with performance on spatial orienting tasks due to the putative role of these regions in self-motion perception and examined whether age is a moderator of this relationship. Given the limited published literature in this space, we also examine and report the effect of age on the activity strength in these OF-sensitive regions during tasks containing navigationally relevant stimuli.

To test this hypothesis, we designed a visual path integration (VPI) fMRI task that highlights the use of visual self-motion information to track one’s position relative to a goal location, which in this case was always the start of each path [42]. Our goal was to emphasize the use of optic flow to compute one’s trajectory in real time. To perform the task at 100% accuracy, participants were required to track their forward motion (during which radial optic flow was experienced) in addition to the direction of each turn taken on the path; exclusively noting the direction of turns taken on each path was not sufficient to perform the task correctly. We also designed a turn counting (TC) fMRI task, which contains the same visual self-motion information as the VPI task, but does not require that participants use it to track their position. In this task, only counting the number of turns, regardless of the direction of the turns, was sufficient to perform at 100% accuracy. Participants performed these tasks in the MRI scanner while we measured their brain activity and performance accuracy. Despite the unrealistic setting of the MRI scanner, these tasks have strong ecological foundations because the stimuli were filmed in a real neighborhood at normal walking speed; they are similar to what participants might experience on a day-to-day basis when out for a walk. As our goal was to test whether OF-sensitive cortical regions are involved in performance on spatial orienting tasks requiring the use of visual self-motion information, we used an optic flow localizer to identify the location of OF-sensitive regions that responded more strongly to coherent radial motion patterns than scrambled motion patterns at the group level in our sample. We verified that these OF-sensitive regions responded to the visual motion present in our tasks and then assessed the relationship between the activity strength in these regions during the VPI task and VPI task performance in a focused subset of regions.

## 2. Materials and Methods

### 2.1. Participants

Data were collected from 51 participants; 29 were young (mean age: 25.2 ± 3.42 years, range: 20–34, 17 female/12 male) and 22 were aged (mean age: 70 ± 4.87 years, range: 62–80, 16 female/6 male). Most participants were recruited from the greater Boston area. All participants had normal or corrected-to-normal vision and lacked major contraindications to MRI. No participants were taking antipsychotic medications. Six aged adults were on stable doses of medication to treat mild anxiety and depression, and one aged adult was taking gabapentin for pain. One young and one aged adult were on stable doses of Adderall. The number of aged adults on psychoactive medications was significantly greater than the number of young adults (c^2^(1, *n* = 51) = 8.09, *p* = 0.0044). All participants completed the same study visit with tasks performed in the same order. The study took place at the Center for Biomedical Imaging, which is located on the Boston University School of Medicine campus. The study was approved by the Institutional Review Board at the Boston University School of Medicine and was conducted in accordance with the Declaration of Helsinki.

All aged participants underwent a screening phone call in which the Modified Telephone Interview for Cognitive Status (TICS-M) [43] was administered in order to assess whether cognition was sufficiently intact. The TICS-M assesses orientation, attention, comprehension, language, memory, and executive function. Aged participants who scored 32 or higher on the TICS-M were eligible to participate. Half of the aged participants had also received a consensus diagnosis of cognitively normal from the Boston University Alzheimer’s Disease Center (ADC) within a year of participating in the study as part of their participation in the ongoing Health Outreach Program for the Elderly (HOPE) study.

### 2.2. Cognitive Assessments

Immediately after on-site screening and consent, aged participants completed the Montreal Cognitive Assessment (MoCA) [44]. All aged participants scored 25 or above on the MoCA (21/22 participants scored 26–30; 1/22 participants scored 25), which is in line with normal cognition for a highly educated sample [44,45,46,47]. Most of the errors were made on delayed recall with 13/22 participants losing points in this area. Out of these 13 participants, 10 remembered all words when given a cue (indicative of difficulty in memory retrieval), and 3 were not able to remember all words when given cues (indicative of difficulty in memory encoding). The following represent the number of participants that lost points in the other areas: abstraction (5/22), visuospatial/executive (5/22), language (3/22), orientation (1/22), naming (1/22). Four participants made no errors on the MoCA.

### 2.3. Functional MRI Paradigms

Prior to entering the MRI scanner, instructions for each task were explained. Participants were shown 5 practice trials of the VPI task (1 zero-turn trial, 2 one-turn trials, 2 two-turn trials, in that order) and 3 practice trials of the turn counting (TC) task. During these practice trials, participants were given the opportunity to ask questions, received performance feedback, and were given the option to view trials more than once if incorrect. Once positioned in the MRI scanner, they were shown an additional 5 practice trials of the VPI task (1 zero-turn trial, 2 one-turn trials, 2 two-turn trials, in that order) to practice performing the task while lying in a supine position and to become familiar with responding with the hand-held device. After VPI task runs and just prior to TC task runs, all participants were shown an additional 3 practice trials of the TC task to remind them of the change in task instructions.

#### 2.3.1. Visual Path Integration Task

**Figure 1:**
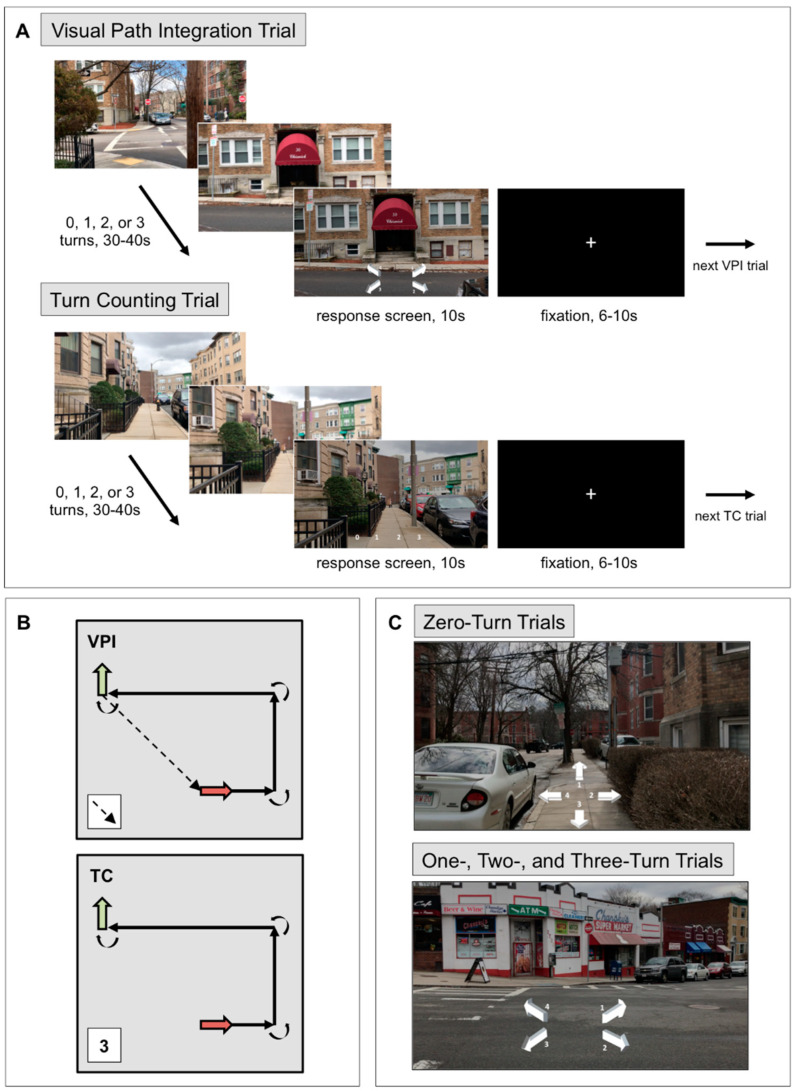
Visual Path Integration and Turn Counting Tasks. (**A**) Schematic showing the structure of a visual path integration (VPI) and turn counting (TC) trial. All trials depicted what participants would view from first-person perspective if they were walking in a neighborhood in Brighton, Massachusetts. (**B**) Birds-eye view of a VPI trial and a TC trial, both of which contained 3 turns. The red arrows show the participant’s starting location and facing direction and the green arrows show the participant’s ending location and facing direction. In the VPI trial, participants were required to keep track of the starting location relative to their position and orientation throughout the path. The correct answer in this particular VPI trial would be arrow 2, which points behind the participant to the right (C, bottom). In a TC trial showing a path with an identical layout, the correct answer would be 3 because that is the number of turns taken in the path. Participants were not required to keep track of their position and orientation in TC trials. (**C**) Response arrows for zero-turn trials (top) had a different configuration from the response arrows for one-, two-, or three-turn trials (bottom). Arrows were numbered and number-arrow pairings were consistent throughout the experiment.

In the VPI task [42], participants used visual stimuli that one would typically experience walking along a short path to keep track of their position and orientation relative to their starting location in each video/trial (see Figure 1 for an illustration of the structure of the task). The VPI task is composed of a series of short videos (30–40 s) filmed from a first-person perspective while walking through a Boston neighborhood. Videos were filmed by the same person (the first author) on the same day using an iPhone 7 at a resolution of 1920 × 1080 pixels at 30 frames/s and a walking pace of 1.7 steps/s, which was maintained with a metronome. Specifically, the individual filming these videos timed her steps according to the metronome, which was set at a speed of 103 beats/min. All sound was removed from these videos. Each trial (i.e., video) showed a path with zero, one, two, or three 90° turns. The VPI task is composed of 4 trials of each type (i.e., zero, one, two, or three turns), which totals 16 trials. All paths are unique in that none were presented to the participant more than one time. At the end of each VPI trial, 4 arrows appeared on the dimmed last frame of the video. Participants were asked to select the arrow that was pointing toward their location at the start of the path relative to their current location and facing direction at the end of the video. They had 10 s to respond, using an MR-compatible device, with the number corresponding to the arrow that they believed was pointing toward the starting location of the path. Responses were balanced within each trial type across response options. Between trials, a white fixation cross on a black background appeared for 6, 8, or 10 s. Participants were shown 2 VPI runs (8 trials per run) and at this point were asked to rate how accurately they believed they performed the task, how difficult the task was for them, and how much effort was required to perform the task on a scale from 1–7. Participants verbally responded to these three questions while in the MRI scanner. Performance on the VPI task was measured as percent correct trials.

#### 2.3.2. Turn Counting Task

After the VPI task, all participants completed the TC task, in which they were asked to count the number of turns but were not required to keep track of their location and orientation (Figure 1). The TC task included 16 videos with zero, one, two, or three 90° turns, just as in the VPI task, but were different videos of different paths. There were 4 trials of each type and trials were balanced across runs. The videos presented in each trial were different paths from those presented in the VPI task, but they were filmed on the same day, in the same neighborhood, by the same person, and with the same parameters as the VPI videos. Again, no path was presented more than once. At the end of each TC trial, 0, 1, 2, and 3 appeared and participants were asked to select the number of turns they believed were made in the video. The VPI and TC tasks are composed of the same type of stimuli, but the way participants are asked to use the visual motion information differs. The VPI task has navigational demands, but the TC task does not; participants were not required to track their location at all during the TC task. After the 2 TC task runs, participants were asked to rate their performance accuracy, the task difficulty, and the effort required, just as they did after the VPI task runs. Performance on the TC task was measured as percent correct trials.

Participants were shown the same set of stimuli (32 videos total, 16 VPI and 16 TC) in the same order. During scanning, all participants first viewed 2 runs of the VPI task (8 videos/trials per run) and then viewed 2 runs of the TC task (8 videos/trials per run). VPI and TC trial types and task runs were not interleaved. We made this experimental choice to minimize confusion over which task was being performed in order to minimize spontaneous path integration during the TC runs.

#### 2.3.3. Optic Flow Localizer

A paradigm consisting of alternating blocks of coherent and scrambled motion [9,48] was used to define OF-sensitive regions of interest (ROIs). The paradigm was generously shared with us by Dr. Marty Sereno at San Diego State University and is freely available (contact msereno@sdsu.edu). The paradigm consists of white dots on a black background that move in alternating 16 s-blocks of coherent motion (dilation, contraction, inward spiral, outward spiral, or rotation) or scrambled motion. In both coherent and scrambled motion blocks, a new field of white dots appears every 500 ms. The type of coherent motion chosen for each 500 ms period is randomly selected from a continuum. In scrambled blocks, the trajectory of each dot is rotated by a random angle, which disrupts the coherent motion of the dot field. A speed gradient is present such that central dots move more slowly than peripheral dots in both coherent and scrambled blocks. Each block was shown 8 times, starting with a coherent block. A red fixation cross was present in the center of the screen throughout and participants were instructed to stay awake and fixate on the cross throughout 2 runs of this task.

### 2.4. Magnetic Resonance Imaging

All participants were scanned at the Center for Biomedical Imaging at the Boston University School of Medicine on a 3T Philips Achieva system with a 32-channel head coil. The one-hour scanning session included the following scans in this order: 2 VPI runs, 2 TC runs, 2 optic flow localizer runs, a 10-min resting-state scan (not used in the analyses in this paper), a fieldmap, and a T1-weighted (T1W) anatomical scan. Axial T2*-weighted scans with blood-oxygenation-level-dependent (BOLD) contrast were acquired during the VPI, TC and optic flow localizer runs (TR/TE: 2000/28 ms, acquired and reconstructed voxel size: 3 × 3 × 3 mm^3^, matrix: 64 × 64, 36 slices, no slice gap, default slice acquisition, EPI factor: 35). Four dummy scans were acquired at the start of each run. For the VPI and TC runs, the number of dynamics varied with the length of each run (~214 dynamics/run). Each optic flow localizer run consisted of 132 dynamics. The axial fieldmap was acquired to correct for distortions in the T2*-weighted images caused by inhomogeneities in the static magnetic field (TR/TE1/TE2: 20/2.3/4.6 ms, acquired voxel size: 2.71 × 2.71 × 3 mm^3^, acquired matrix: 96 × 80, reconstructed voxel size: 1.02 × 1.02 × 3 mm^3^). A sagittal MP-RAGE scan was used to acquire T1 W data for use in image registration (TR/TE: 6.7/3.1 ms, flip angle: 9°, acquired voxel size: 1.11 × 1.11 × 1.2 mm^3^, acquired matrix: 244 × 227, reconstructed voxel size: 1.05 × 1.05 × 1.2 mm^3^, reconstructed matrix: 256 × 256, 140 slices).

The visual stimuli were displayed on an MR-compatible LCD screen (Cambridge Research Systems, BOLDscreen 3 D LCD for fMRI, active area: 50.9 × 29.0 cm^2^) and viewed through a mirror (15.2 × 7.6 cm^2^) mounted on the headcoil approximately 13 cm from the participants’ eyes and 102 cm from the LCD screen. VPI and TC runs were presented in EPrime v2.0 on a PC running Windows 7 Professional. The optic flow localizer was presented on a MacBook Pro (Apple Computer Inc, Cupertino, California, 13-inch Retina display, Early 2015, running High Sierra v10.13.3). Lights in the scanner room were dimmed during the experiment. Participants responded to each VPI and TC trial with their dominant hand using a Current Designs PYKA response pad (Current Designs Inc. Philadelphia, Pennsylvania) in the scanner. Participants with corrected vision but no contact lenses used MRI-compatible glasses for the scanning session. The lens strength that made the paradigms most clear (as reported by the participant) in the MRI scanner was used. After completion of MRI scanning, participants filled out a post-scan questionnaire including questions about VPI task strategy and neighborhood familiarity, among others, outside of the scanner in the CBI lobby.

### 2.5. Global Motion Coherence Thresholds

After the scanning session, global radial and translational motion coherence thresholds were measured using random dot kinematograms. Radial global motion coherence thresholds represented participants’ sensitivity to inward/outward radial optic flow; outward radial optic flow consistent with forward self-motion was experienced while moving forward in the VPI and TC tasks. Translational global motion coherence thresholds represented participants’ sensitivity to left/right translational optic flow, which was experienced during turns in the VPI and TC tasks. Radial optic flow thresholds were used in the analyses presented in this paper because the majority of the motion experienced in the VPI and TC tasks was radial flow. The random dot kinematogram paradigms were generously shared with us by Dr. Peter Bex and were adapted for use in this study.

The random dot kinematogram paradigms were run on a Lenovo computer (Lenovo Inc, Quarry Bay, Hong Kong) with an Intel Core i7-6700 Processor running Windows 10 (Microsoft Inc, Redmond, Washington). Paradigms were executed in MATLAB R2017a in Psychophysics Toolbox version 3.0.14 beta (Mathworks Inc., Natick, Massachusetts). The paradigms were displayed on a Dell E2715H monitor with 1920 × 1080 resolution and a 60 Hz refresh rate. All participants completed tests to measure radial and translational global motion coherence thresholds, which are described below. Participants first completed practice trials of the radial and translational tests to ensure that they understood how to respond, and then they completed the radial test followed by the translational test. Participants completed these tests in a dark room at a viewing distance of 50 cm. They responded with their right hand using left and right clicks to indicate inward and outward motion, respectively, or left and right motion, respectively.

In the radial and translational tests, 200 white dots (diameter = 0.3 degrees, Michelson contrast = 1.0) were presented on a gray screen in each trial. The stimuli subtended a circular area with a diameter of 34 degrees in the center of the screen and a green dot was present in the center. In the radial test, varying proportions of the dots moved inward toward the central green dot or outward away from it. In the translational test, varying proportions of the dots moved to the left or to the right. In each trial, dots appeared for 0.5 s, moved at a speed of 6 degrees/s, and had a lifetime of 3 frames. On the first trial of every test, 100% of the dots moved coherently. If a correct response was provided on a given trial, the proportion of dots moving coherently in that trial was divided by 1.122. If an incorrect response was provided on a given trial, the proportion of dots moving coherently in that trial was multiplied by 1.259. These step sizes were used in order to achieve staircase convergence at 80% correct performance [49]. Participants completed 80 trials of each test. Radial and translational thresholds were calculated by averaging the proportion of dots that were signal (i.e., coherently moving) across reversals in each test (excluding the first 4 reversals). Participants performed these tests with their corrective lenses if their vision was corrected.

### 2.6. Image Processing

All data were exported from the MRI scanner in FSL-NIfTI format and visually inspected prior to use. FMRI data were processed in FSL (FMRIB Software Library, FSL, Oxford, United Kingdom) v5.0.11 [50] using the FMRI Expert Analysis Tool (FEAT) v6.00.

#### 2.6.1. Preprocessing

Motion correction with MCFLIRT, B0 unwarping, spatial smoothing with a FWHM of 6 mm, and highpass temporal filtering with a cutoff of 90 s were carried out. To clean the data, a single-session independent components analysis (ICA) was run on each optic flow localizer run, VPI run, and TC run. Components were classified by hand as noise or signal according to their spatial pattern, timecourse, and frequency spectrum, and noise components were regressed out of the data [51]. FMRI data were linearly registered to each participant’s T1W image using boundary-based registration [52,53,54], and each participant’s T1W image was nonlinearly registered to the MNI152 2 mm atlas using 12 degrees of freedom and a 2 mm warp resolution [55]. FSL combines these transformations into a single step to transform fMRI data into MNI152 space. All image registrations were visually inspected to ensure accuracy.

#### 2.6.2. Defining OF-Sensitive ROIs

**Figure 2:**
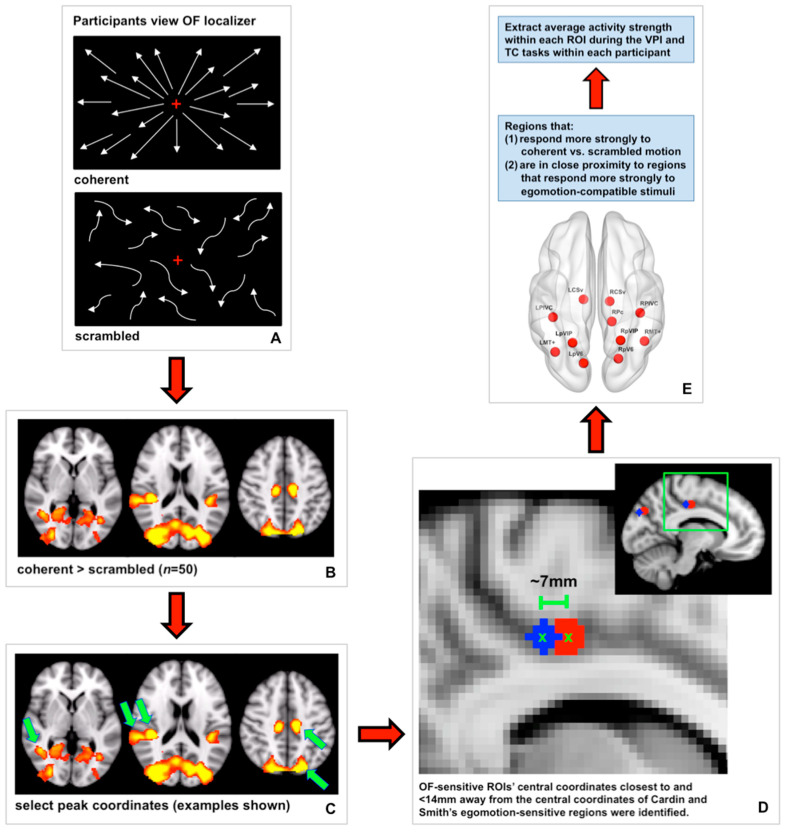
Defining Optic Flow-Sensitive Regions of Interest. (**A**) Participants viewed an optic flow localizer in which blocks of coherent radial dot motion alternated with blocks of scrambled dot motion while their brain activity was measured using blood-oxygenation-level-dependent (BOLD) fMRI. (**B**) Regions that responded more strongly during coherent blocks versus scrambled blocks were mapped at the group level. (**C**) Coordinates corresponding to peaks of activity within these group maps were identified and by hand. (**D**) The Euclidean distance between the coordinates corresponding to these peaks of activity and the average center coordinates of regions shown to respond more strongly to egomotion-compatible versus egomotion-incompatible stimuli [8] were measured. Regions whose center coordinates were < 14 mm away from Cardin and Smith’s regions were selected and named for the region to which they were closest. The figure inset shows areas LpV6 (parieto-occipital sulcus) and LCSv (cingulate sulcus), which is also shown in the magnified image. The regions shown in blue correspond to the average coordinates of Cardin and Smith’s regions and the regions shown in red correspond to our OF-sensitive regions. (**E**) The final set of 11 OF-sensitive regions is shown on a cortical surface image created in Brain Net Viewer v1.6 [56]. The average parameter estimate within each of these regions was extracted in each participant during both the VPI and the TC tasks as a measure of activity strength.

Many cortical regions have been reported to be responsive to motion, specifically coherent motion, and have different degrees of selectivity for different types of motion (for examples see [7,8,9,19]). In an effort to focus our analyses, we identified OF-sensitive regions for our analyses using two criteria (see Figure 2 for a schematic). First, we defined a set of regions that responded more strongly to coherent radial dot motion (i.e., global motion patterns) relative to scrambled dot motion (i.e., local motion patterns) at the group level. To do this, the preprocessed data collected during the optic flow localizer from 50/51 young and aged participants were prewhitened. The optic flow localizer was not shown to one aged participant who reported experiencing some motion sickness after the VPI and TC runs. To determine where brain activity was increased during coherent dot motion compared to scrambled dot motion, a double-gamma HRF convolution was applied to the stimulus waveform representing coherent dot motion. Fixed-effects analyses were used for within-subject higher-level analyses. To create a group map showing where brain activity was significantly increased during coherent motion relative to scrambled motion at the group level, FSL’s FLAME 1 + 2 was used with a Z threshold of 3.1 and a FWER-corrected cluster *p* threshold of 0.05. Coordinates corresponding to peaks of activity within this group map were identified by hand.

**Figure 3:**
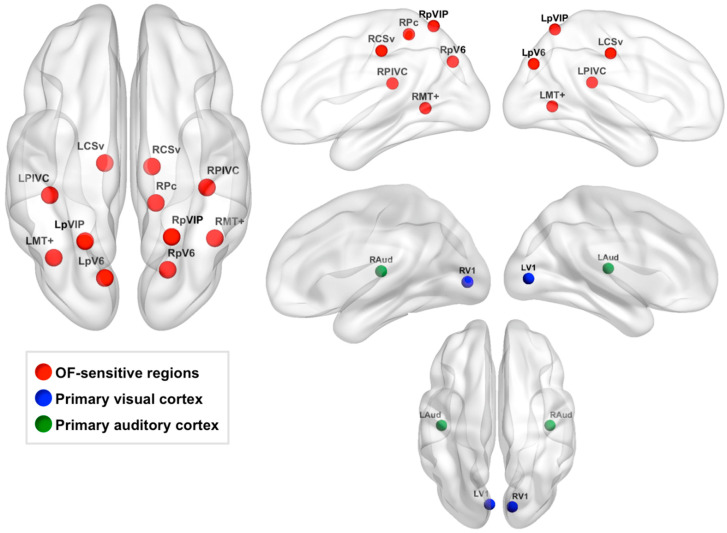
Optic Flow-Sensitive and Control Regions of Interest. OF-sensitive cortical regions were defined (see Figure 2) and are shown in red. We defined two types of control regions. Our negative control regions were left and right primary auditory cortex, which are shown in green. These were defined by hand in Heschl’s gyrus in the left and right hemisphere using the Harvard-Oxford Cortical Structural Atlas. Our other control regions were left and right primary visual cortex, which are shown in blue. Primary visual cortex responds to visual stimuli with and without motion, but does not show a preference for coherent motion, which is associated with global motion patterns, versus scrambled motion, which is associated with local motion patterns. Cortical surface images were created using Brain Net Viewer v1.6 [56].

Second, we selected a subset of these regions based on their proximity to regions sensitive to “egomotion-compatible” stimuli reported by Cardin and Smith [8]: middle temporal complex (MT+), putative area V6 (pV6), parietoinsular vestibular cortex (PIVC), putative ventral intraparietal area (pVIP), putative area 2v (p2v), cingulate sulcus visual area (CSv), and a region in the precuneus (Pc) in the ascending ramus of the cingulate sulcus. We reasoned that regions that respond more strongly to egomotion-compatible stimuli would be most relevant to our analyses due to our focus on using visual self-motion cues to path integrate. To identify the regions identified by our optic flow localizer that corresponded to the egomotion-sensitive regions reported in Cardin and Smith [8], we measured the Euclidean distance between the center coordinates of Cardin and Smith’s regions and the center coordinates of our OF-sensitive regions (Figure 2). OF-sensitive regions whose center coordinates were within 14 mm of any of Cardin and Smith’s egomotion-compatible regions were selected for further analysis and named according to the region to which they were closest. The accuracy of this process was assessed by viewing each region on the MNI152 2 mm atlas after naming to ensure regions were located in an appropriate anatomical area. This process resulted in our definition of the following 11 OF-sensitive regions: left and right CSv (LCSv and RCSv), left and right MT+ (LMT+ and RMT+), left and right PIVC (LPIVC and RPIVC), left and right pV6 (LpV6 and RpV6), left and right pVIP (LpVIP and RpVIP), and right Pc (RPc) [8,19]. The center coordinates of these regions are listed in Table 1, and the regions are displayed on a cortical surface in Figure 3.

#### 2.6.3. Defining Control ROIs

Primary auditory cortex ROIs in the left and right hemisphere were defined as negative control regions. We reasoned that activity in primary auditory cortex should not be relevant to our tasks or global visual motion perception. To define these ROIs, we used the Harvard-Oxford Cortical Structural Atlas included in FSLview to select the center coordinates of these regions in the left and right hemisphere, abbreviated as LAud and RAud, respectively. Specifically, these coordinates were located in left and right Heschl’s gyri (MNI152 coordinates: LAud: (−44, −20, 10), RAud: (42, −20, 10)). Binary spheres with a radius of 5 mm were centered at these coordinates. These regions are shown in green in Figure 3.

We also defined V1 ROIs in the left and right hemisphere as control regions. V1 responds more to local motion than global motion [7] and is therefore an area that has not been strongly implicated in self-motion perception, which relies on accurate perception of global motion patterns. However, because V1 relays visual motion information to higher-level global motion areas, we created these control regions to assess whether (1) global radial motion thresholds were associated with V1 activity during VPI similarly to our global motion areas and (2) whether VPI performance was associated with activity strength in V1 during VPI. We carried out these analyses to assess whether our findings were at all associated with local motion processing or whether they were specific to global motion processing. In contrast to our primary auditory cortex ROIs, we anticipated that our task would evoke strong V1 activity, and it did. To define our V1 ROIs, we extracted the thresholded V1 ROIs [57] from FreeSurfer v6.0 (http://surfer.nmr.mgh.harvard.edu/ (accessed on 27 March 2019), Athinoula A. Martinos Center for Biomedical Imaging, Cambridge, Massachusetts) in MNI152 2 mm space. We then overlaid these ROIs on a group-level map of significant brain activity during VPI relative to rest. Coordinates in the left and right hemisphere where peak activity during VPI overlapped well with the V1 FreeSurfer ROIs were selected by hand in the left and right hemisphere (MNI152 coordinates: LV1: (−6, −82, 2), RV1: (12, −84, 2)). Spheres with a radius of 5 mm were centered at these coordinates. These regions are shown in blue in Figure 3.

#### 2.6.4. Measuring Brain Activity in ROIs during the VPI and TC Tasks

To measure brain activity during VPI and TC, the pre-processed data from each task were prewhitened and a double-gamma HRF convolution was applied to the stimulus waveform representing the VPI or TC periods in each run. The regressors for VPI and TC did not include the 10-s response period. The response period was grouped with the fixation cross period, and thus, only the active VPI and TC periods were modeled. Fixed-effects analyses were used for within-subject higher-level analyses to create maps representing where brain activity was increased during VPI or TC relative to baseline (white fixation cross on black background) within each participant. The average parameter estimate within each OF-sensitive region was extracted and used as a measure of the average change in brain activity during VPI or TC relative to baseline within each OF-sensitive region in each participant. The average parameter estimate within LAud, RAud, LV1, and RV1 was extracted and used as a measure of the average change in brain activity relative to baseline during VPI or TC within each of these regions in each participant.

To evaluate whether OF-sensitive regions were responding to motion in the VPI and TC tasks, we measured whether the average response within each OF-sensitive region was greater than zero during the VPI and TC tasks. One-tailed t-tests were performed to assess this. We anticipated that activity in LV1 and RV1 would be greater than zero during our tasks and tested this with one-tailed t-tests, as well. For comparison, we tested whether the average response within LAud and RAud differed from zero during the VPI and TC tasks using one-tailed t-tests. These and all subsequent statistical analyses were performed in JMP Pro v13.

### 2.7. Effect of Age on VPI and TC Task Performance

Age was treated as a binary variable in all analyses. Wilcoxon tests were used to compare VPI accuracy, TC accuracy, and all self-report measures related to the VPI and TC tasks between groups either because the data were skewed or the variables were ordinal in nature. Neighborhood familiarity was coded as a binary variable (i.e., yes, familiar/no, not familiar) and a contingency test was used to determine (1) whether this measure differed between groups and (2) whether the fraction of participants who reported that this familiarity did not help them differed between groups. Most participants in both groups endorsed using an “updating” strategy in the VPI task in which they described keeping the starting location in mind and updating it throughout each trial and particularly during the turns experienced in each path. A contingency test was used to determine whether the number of participants that endorsed this strategy differed between groups.

### 2.8. Assessing the Effects of Age and Performance on OF-Sensitive Region Activity

To further focus our analyses on a subset of the 11 OF-sensitive regions defined, we performed linear correlations to determine whether global radial motion thresholds were related to the activity strength within any OF-sensitive regions during VPI. We chose to assess relationships between activity strength and global radial motion thresholds because participants overwhelmingly experienced radial motion during the VPI task. Given the block design fMRI paradigm, the activity strength measured was largely reflective of radial motion. Translational motion was only briefly experienced during turns, and therefore, the activity strength measured within OF-sensitive regions in this experiment would not be expected to reflect global translational motion thresholds.

Activity within four OF-sensitive regions during VPI showed significant inverse relationships with radial global motion thresholds: RCSv (r(48) = −0.303, *p* = 0.0307), RMT+ (r(48) = −0.341, *p* = 0.0144), LpVIP (r(48) = −0.337, *p* = 0.0155), and RpVIP (r(48) = −0.31, *p* = 0.0268). Activity within LMT+ during VPI showed an inverse relationship with radial global motion thresholds that reached trend level (r(48) = −0.257, *p* = 0.0687). Though this relationship reached trend-level significance, the significant relationship found between activity strength in RMT+ during VPI and radial global motion perception and MT+’s demonstrated role in motion perception led us to include LMT+ in our focused subset. These relationships remained when controlling for age. Inverse relationships were found between radial motion thresholds and activity during VPI for all 11 OF-sensitive regions; those with higher radial motion thresholds tended to have weaker activity in OF-sensitive regions during VPI. This pattern of brain activity is fitting considering that higher global motion thresholds are taken to represent weaker sensitivity to global motion patterns. No significant or trend-level relationships were present between radial global motion thresholds and activity within LAud, RAud, LV1, or RV1 during VPI. We examined whether there was an effect of age on activity in RCSv, LMT+, RMT+, LpVIP, or RpVIP during VPI or during TC using two-tailed t-tests.

Next, we examined whether there was a relationship between performance on the VPI task and activity within OF-sensitive regions during VPI and whether age moderated this relationship. Regression models in which VPI accuracy was the dependent variable and activity in RCSv, LMT+, RMT+, LpVIP, or RpVIP during VPI, age, and an interaction term were the independent variables were constructed. A separate model was constructed for each region, and we constructed models with activity measured in LAud, RAud, LV1, and RV1 as negative controls. As inverse relationships between radial global motion thresholds and activity in our OF-sensitive regions were found, we predicted positive relationships between OF-sensitive region activity strength during VPI and VPI accuracy. For regions in which a significant relationship between OF-sensitive region activity during VPI and VPI accuracy were found, identical models were built using activity in those regions measured during TC to assess the specificity of the relationships to a task with navigational demands. For regions in which a significant interaction between age and activity strength were found, linear correlations and one-tailed tests predicting positive relationships were performed in the young and aged groups separately.

Lastly, we used stepwise variable selection to create a model predicting VPI accuracy using activity strength within OF-sensitive regions during VPI to explore whether activity in a combination of regions might predict accuracy better than activity within individual regions. Activity strength in RCSv, LMT+, RMT+, LpVIP, and RpVIP during VPI were entered into stepwise variable selection. Stepwise variable selection was performed to minimize the Bayesian Information Criterion with forward selection and backward selection. Variables selected for the model were tested for collinearity. A second model was built using activity strength during TC within the OF-sensitive regions selected for the VPI model to determine whether the relationship between activity and performance shown in the VPI model was also present for activity measured during the TC task. Standard least squares regression models were used.

## 3. Results

### 3.1. Effect of Age on VPI and TC Task Performance

Table 2 shows participant demographics and the mean, standard deviation, and range for the VPI and TC task performance measures for the young and aged groups as well as the measures that were significantly different between the young and aged groups.

Aged participants performed significantly worse on the VPI task than young participants (VPI Accuracy: t(22.8) = −3.35, *p* = 0.0028), but both groups performed equally well on the TC task. Reflecting this, the aged group reported significantly lower accuracy, greater difficulty, and greater effort exerted on the VPI task (but not the TC task) relative to the young participants (Perceived VPI Accuracy: t(49) = 2.63, *p* = 0.0114; Perceived VPI Difficulty: t(49) = 4.46, *p* < 0.0001; Perceived VPI Effort: t(49) = 4.44, *p* < 0.0001). None of these differences were explained by differences in psychoactive medication use between groups. In the aged group, all but one participant got 3/4 (4/22) or 4/4 (17/22) zero-turn trials correct, which provides confidence that most participants understood the VPI task and were paying attention. The participant that responded incorrectly to all four zero-turn trials performed poorly on the VPI task (43.8% correct), but performed well on the TC task (75% correct) suggesting that this participant was selectively disoriented when navigational demands were present.

Most participants in both groups (young: 24/29, aged: 18/22) described their VPI strategy as one that involved keeping the starting location in mind throughout the path and updating it, especially with each turn. Additional strategies included envisioning the path/route as if it were being traced on a map or GPS system (young: 4/29, aged: 1/22), keeping track of the number of left/right turns (young: 0/29, aged: 2/22), comparing visual cues at the start and end of the path (young: 0/29, aged: 1/22), and unclear (young: 1/29, aged: 0/22). The number of participants endorsing the updating strategy did not differ between groups. We did not test whether the number of participants endorsing the other strategies differed between groups due to the small numbers of participants endorsing those strategies.

Lastly, participants were asked to report whether the neighborhoods in the VPI and TC tasks were familiar to them and approximately half the young and half the aged participants reported the neighborhoods were familiar. The number of participants reporting that the neighborhoods were familiar did not differ between groups. Of those whom reported the neighborhoods were familiar, 76.5% of young participants and 90.9% of aged participants reported that this was *not* helpful to them while performing the task. In fact, 6 young participants and 3 aged participants specifically reported the familiarity of the neighborhoods to be distracting in performing the VPI task. Within the aged group, familiarity did not have a significant effect on VPI accuracy.

### 3.2. OF-Sensitive Region Activity Strength during VPI and TC Tasks

**Figure 4:**
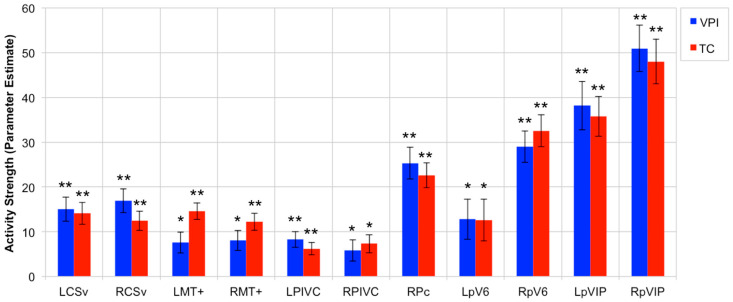
Activity within OF-Sensitive Regions during the VPI and TC Tasks. Activity within all OF-sensitive regions during the VPI and TC tasks was significantly greater than zero relative to baseline in our sample of young and aged adults (*n* = 51). Activity within primary visual cortex ROIs, LV1 and RV1 (Figure S1), was also significantly greater than zero and had an average activity greater than that of RpVIP during both tasks. * *p* < 0.01, ** *p* < 0.0001.

Figure 4 shows the average activity strength within the 11 OF-sensitive regions defined using the optic flow localizer and as having an increased response to egomotion-compatible stimuli according to Cardin and Smith [8]. The average activity in all 11 regions was significantly greater than zero during VPI and TC in the young and aged participants (LCSv, VPI: t(50) = 5.57, *p* < 0.0001; LCSv, TC: t(50) = 5.8, *p* < 0.0001; LMT+, VPI: t(50) = 3.23, *p* = 0.0011; LMT+, TC: t(50) = 7.95, *p* < 0.0001; LPIVC, VPI: t(50) = 4.71, *p* < 0.0001; LPIVC, TC: t(50) = 4.44, *p* < 0.0001; LpV6, VPI: t(50) = 2.85, *p* = 0.0032; LpV6, TC: t(50) = 2.69, *p* = 0.0048; LpVIP, VPI: t(50) = 7.04, *p* < 0.0001; LpVIP, TC: t(50) = 8.08, *p* < 0.0001; RCSv, VPI: t(50) = 6.37, *p* < 0.0001; RCSv, TC: t(50) = 5.8, *p* < 0.0001; RMT+, VPI: t(50) = 3.58, *p* = 0.0004; RMT+, TC: t(50) = 6.42, *p* < 0.0001; RPc, VPI: t(50) = 7.12, *p* < 0.0001; RPc, TC: t(50) = 8.09, *p* < 0.0001; RPIVC, VPI: t(50) = 2.46, *p* = 0.0086; RPIVC, TC: t(50) = 3.60, *p* = 0.0004; RpV6, VPI: t(50) = 8.34, *p* < 0.0001; RpV6, TC: t(50) = 9.09, *p* < 0.0001; RpVIP, VPI: t(50) = 9.86, *p* < 0.0001; RpVIP, TC: t(50) = 9.60, *p* < 0.0001). Figure S1 shows the average activity strength within LAud, RAud, LV1, and RV1 during VPI and TC. In contrast to the OF-sensitive regions, the average activity in LAud during VPI was significantly less than zero (t(50) = −2.73, *p* = 0.0044) whereas the activity in LAud during TC and RAud during VPI and TC did not differ from zero. The average activity in LV1 and RV1 was significantly greater than zero, as expected (LV1, VPI: t(50) = 14.7, *p* < 0.0001; LV1, TC: t(50) = 13.84, *p* < 0.0001; RV1, VPI: t(50) = 18.4, *p* < 0.0001; RV1, TC: t(50) = 18.2, *p* < 0.0001).

### 3.3. Effect of Age on OF-Sensitive Region Activity during VPI and TC

**Figure 5:**
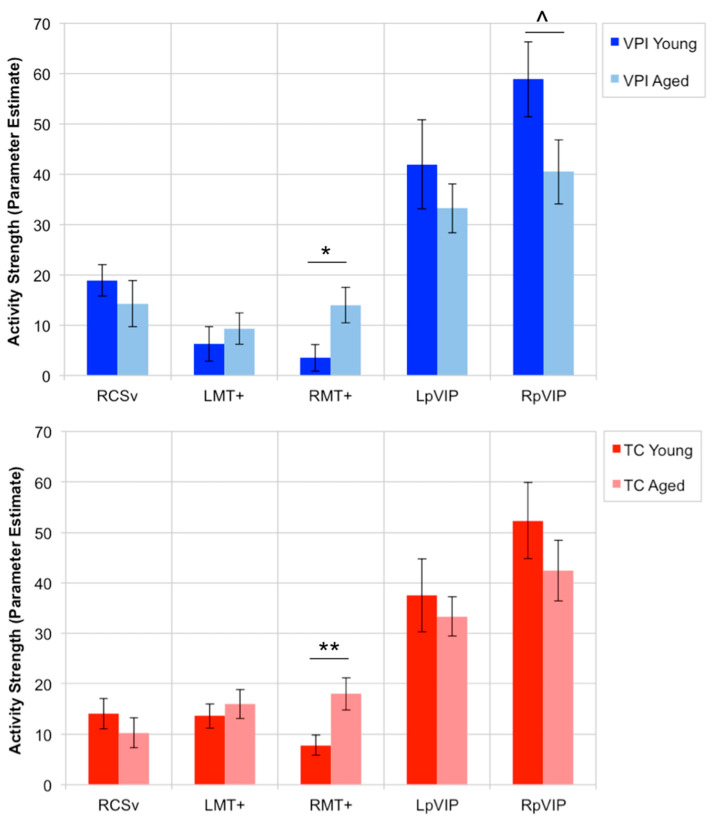
Effect of Age on Activity within Subset of OF-Sensitive Regions during VPI and TC. Analyses examining the effect of age and performance on activity within OF-sensitive regions were performed on regions whose activity strength during VPI was inversely associated with radial global motion threshold. There were significant effects of age on activity within RMT+ during both the VPI and TC tasks with aged adults showing stronger activity. A trend for stronger activity in RpVIP during the VPI task in young adults was present. ^ *p* < 0.08, * *p* < 0.05, ** *p* < 0.01.

The rest of the results only consider the following OF-sensitive regions whose activity strength during VPI showed an inverse relationship with global radial motion threshold: RCSv, LMT+, RMT+, LpVIP, and RpVIP. The only significant age effect that was found was that RMT+ activity during both the VPI task (t(49) = 2.43, *p* = 0.0187) and the TC task (t(49) = 2.81, *p* = 0.007) was greater in aged adults compared to young adults. This difference was not explained by a difference in use of psychoactive medications between groups. There was a trend for greater activity in RpVIP in young adults during VPI (t(49) = −1.80, *p* = 0.0781) that was not present during TC (t(49) = −0.984, *p* = 0.33). Figure 5 shows the average activity strength within this set of ROIs in young and aged adults during the VPI and TC tasks.

### 3.4. Relationship between VPI Accuracy and OF-Sensitive Region Activity Strength

All regression models predicting VPI accuracy showed a significant effect of age on performance, reflecting the results already presented showing that aged adults performed worse on the VPI task. Significant positive effects of activity strength in LMT+ (β = 0.429, t(47) = 3.45, p_FDR_ = 0.00178), LpVIP (β = 0.256, t(47) = 3.60, p_FDR_ = 0.00085), and RpVIP (β = 0.164, t(47) = 2.63, p_FDR_ = 0.0172) during VPI and VPI accuracy were found. The interaction effects within these three models were significant as well (LMT+ × age: β = −0.383, t(47) = −3.08, p_FDR_ = 0.00345, LpVIP × age: β = −0.256, t(47) = −3.60, p_FDR_ = 0.00085, RpVIP × age: β = −0.151, t(47) = −2.42, p_FDR_ = 0.0195).

To assess these relationships further, linear correlations predicting positive relationships between activity in LMT+, LpVIP, and RpVIP during VPI and VPI accuracy were performed within young and aged groups separately. In aged adults, significant positive relationships between activity in LMT+ (r(20) = 0.518, *p* = 0.0068), LpVIP (r(20) = 0.513, *p* = 0.0073), and RpVIP (r(20) = 0.417, *p* = 0.0267) during VPI and VPI accuracy were found. In young adults, these relationships were weaker and did not reach significance: LMT+ (r(27) = 0.157, *p* = 0.208), LpVIP (r(27) = 0.0021, *p* = 0.496), RpVIP (r(27) = 0.0974, *p* = 0.308). We assessed whether activity strength in LMT+, LpVIP, or RpVIP during TC and their interactions with age were significant predictors of VPI performance using identical models with the exception that activity strength in these regions was measured during TC. There was a positive effect of activity in LpVIP during TC on VPI performance (β = 0.263, t(47) = 2.86, p_FDR_ = 0.00732) and a significant interaction (LpVIP × age: β = −0.258, t(47) = −2.80, p_FDR_ = 0.00732) with a stronger relationship between LpVIP activity during TC and VPI accuracy in aged adults (r(20) = 0.423, *p* = 0.0248) compared to young adults (r(27) = 0.0398, *p* = 0.419). There were no significant effects of activity in LAud, RAud, LV1, or RV1 during VPI on VPI performance, and no significant interactions. Furthermore, in exploratory analyses, no significant effects of activity strength in regions that have been shown to be important to spatial navigation processes (hereafter referred to as canonical navigation regions) on VPI performance and no significant interactions were found (Supplementary Material [58,59,60,61,62,63,64,65,66,67,68,69,70]).

The multiple regression model predicting VPI accuracy was built using data only from the aged group because the relationships between activity in OF-sensitive regions and performance were stronger in this group compared to the young group. Both forward and backward stepwise variable selection identified activity in LMT+ and RpVIP during VPI as variables for the model predicting VPI accuracy in the aged group. LMT+ and RpVIP activity during VPI were not correlated (r(20) = 0.0502, *p* = 0.824). The overall model was significant (R^2^ = 0.421, F(2,19) = 6.92, *p* = 0.0055), and both LMT+ and RpVIP activity during VPI were significant predictors of VPI accuracy (LMT+, VPI: β = 0.780, t(19) = 2.85, p_FDR_ = 0.0205; RpVIP, VPI: β = 0.297, t(19) = 2.25, p_FDR_ = 0.0368). The model predicting VPI accuracy using LMT+ and RpVIP activity measured during TC was not significant (R^2^ = 0.139, F(2,19) = 1.54, *p* = 0.241) and neither variable was a significant predictor of VPI accuracy within this model (LMT+, TC: β = 0.602, t(19) = 1.64, p_FDR_ = 0.233; RpVIP, TC: β = 0.076, t(19) = 0.44, p_FDR_ = 0.665). Psychoactive medications did not have an effect on VPI accuracy or the activity strength within LMT+ or RpVIP during VPI. Table 3 shows the details of each model.

## 4. Discussion

The results of these experiments show that activity strength within OF-sensitive cortical areas during a visual path integration task is a factor associated with performance accuracy on that task in aged adults. To our knowledge, this is the first direct demonstration of such a relationship and first exploration of this idea using fMRI. Similar to what many other spatial navigation and path integration studies have found, the aged adults performed worse than the young adults on the VPI task, which required using visual motion cues to keep track of location and orientation, but not on the TC task, which contained the same visual motion cues but did not require participants to use them for spatial orientation.

Our set of 11 OF-sensitive regions was defined based on their stronger response to coherent versus scrambled motion and their reported sensitivity specifically to egomotion-compatible stimuli. Accordingly, all 11 regions responded to the motion in our VPI and TC tasks, and the activation strength during VPI in 5 of these regions showed an inverse relationship with radial global motion threshold. Participants who were less sensitive to radial global motion (i.e., had higher perceptual thresholds) had weaker activity within RCSv, LMT+, RMT+, LpVIP, and RpVIP during the VPI task. Focusing on this set of regions, aged adults showed stronger activity in RMT+ during both the VPI and the TC tasks relative to young adults. No other significant effects of age on activity strength were found.

Stronger activity in LMT+, LpVIP, and RpVIP during VPI was associated with higher accuracy on the VPI task in aged adults, and the relationships involving LMT+ and RpVIP activity were specific to activity measured during VPI. There were no significant relationships between activity strength in LAud or RAud during VPI and VPI performance, as would be expected for activity in auditory cortex. Additionally, there were no significant relationships between activity strength in LV1 or RV1 during VPI and VPI performance, suggesting that the relationships seen in in LMT+, LpVIP, and RpVIP are more closely associated with global motion processing rather than local motion processing. Lastly, a linear regression model with activity strength in LMT+ and RpVIP during VPI predicted 42% of the variance in VPI accuracy in aged adults. The equivalent model using activity in these regions during TC as predictors was not significant. Overall, these results demonstrate a link between sensitivity to global radial motion patterns, activity strength within visual motion areas during visual path integration, and visual path integration accuracy, particularly in aged adults.

### 4.1. Activity in RCSv, LMT+, RMT+, LpVIP, and RpVIP during VPI Was Inversely Related to Global Radial Motion Thresholds

Our main goals in this study were to examine the effect of age on activity strength in OF-sensitive regions during real-time visual path integration and whether activity strength in these regions was associated with visual path integration performance. We focused on OF-sensitive regions because of their implication in visual self-motion perception; this is central to our VPI task and is present in our TC task, but it is not essential to TC task performance. We defined a set of 11 OF-sensitive regions that (1) responded more strongly to coherent dot motion than scrambled dot motion and (2) were in close proximity to regions that have been reported to respond more strongly to egomotion-compatible than egomotion-incompatible stimuli. Activity within a subset of the 11 OF-sensitive regions (LMT+, RMT+, LpVIP, RpVIP, and RCSv) measured during the VPI task showed an inverse relationship with global radial motion thresholds measured outside of the MRI scanner. We used this criterion to further focus our analyses on OF-sensitive regions whose activity during VPI was most closely associated with perceptual sensitivity to global radial motion patterns, which are experienced during self motion.

The approach described above led us to focus our analyses on a set of OF-sensitive regions that fMRI studies in humans and electrophysiological and fMRI studies in macaques have implicated in self-motion and heading perception. Activity measured in OF-sensitive regions LMT+ and RMT+ during VPI was inversely associated with radial global motion sensitivity. The notation MT+ is frequently used in human fMRI studies to refer to the combination of MT, MST, and other motion-sensitive areas within this vicinity [16,18]. Human fMRI studies have shown that MST and the broader area MT+ are sensitive to heading direction based solely on optic flow [20,22,23], similar to the properties of MST in macaques noted in the introduction. Research in both species strongly implicates MST/MT+ in self-motion perception [12,13,14].

Activity measured in OF-sensitive regions LpVIP and RpVIP during VPI was inversely associated with radial global motion sensitivity, as well. In humans, several visual motion-sensitive areas, including regions that have been shown to be more sensitive to coherent than scrambled motion, have been identified in the vicinity of the intraparietal sulcus, in which LpVIP and RpVIP are located [71]. FMRI studies have also shown that regions within the intraparietal sulcus are sensitive to heading direction based on optic flow [21,22]. Our left and right pVIP ROIs are putative human homologues of macaque area VIP. We emphasize the putative homology of this region more than that of MST because the intraparietal sulcus and surrounding regions differ quite a bit between humans and macaques [72,73]. Our pVIP regions correspond closely with the average coordinates of pVIP reported in Cardin and Smith [8], though both regions, in addition to the heading-sensitive region reported by Peuskens and colleagues [22], appear to be located dorsal to the fundus of the intraparietal sulcus. Although the correspondence between pVIP and macaque VIP is less certain than the correspondence between MT+ and MST, regions in the vicinity of our pVIP are nevertheless implicated in self-motion processing in humans.

We were also able to identify LCSv and RCSv, and activity measured in RCSv during VPI was inversely associated with radial motion thresholds. In humans, CSv has been shown to be sensitive to heading direction based on visual flow information [21] and to respond to real motion versus motion generated from retinal motion (i.e., the region can differentiate between retinal motion due to self-motion versus eye movement accounts) [74]. Our ability to contextualize CSv in the macaque literature is limited, particularly because this region was identified first in humans [19] and only recently has a potentially homologous region been identified in the macaque using imaging and egomotion-compatible stimuli [10]. The properties of CSv derived from fMRI studies suggest a role for the region in self-motion perception.

Taken together, our ability to define our set of OF-sensitive areas based on their increased response to coherent versus scrambled motion and the response of the OF-sensitive regions to the motion in our task are in line with the human and non-human primate literature on the role of these regions in self-motion perception. Narrowing down this set of regions based on the relationship between activity strength in these regions during VPI and radial motion perceptual thresholds led us to select L/RMT+, L/RpVIP, and RCSv for further analysis. The literature provides strong support for roles of MST/MT+ and VIP/pVIP in self-motion perception and heading. The literature is consistent with CSv playing a role in self-motion perception and heading, but more study is needed to better understand its role.

### 4.2. Stronger Activity in RMT+ in Aged Adults during VPI and TC

After narrowing down our set of 11 OF-sensitive regions to 5 whose activity during VPI was inversely associated with global radial motion sensitivity, we examined the effect of age on activity in these 5 regions during the VPI and TC tasks. Aged adults had stronger activity in RMT+ during both the VPI and TC tasks, showing that age had a similar effect on the response of this region to motion regardless of the presence of navigational demands in the task. To our knowledge, only one published study has directly investigated the effect of aging on activation within MT+ in response to radial motion in humans using fMRI [75]. In whole-brain analyses, the authors found significantly stronger activity in a cluster in the vicinity of RMT+ in aged adults compared to young adults while they viewed radial dot motion that was not found in the left hemisphere. This aligns with our finding of increased RMT+ activity during the VPI and TC tasks, both of which contained radial coherent motion, in aged adults.

What might explain the increased response in MT+ during VPI and TC in aged adults? Electrophysiological research in young and aged macaques has examined the effects of aging on the speed and direction tuning of MT neurons, which provide input to MST. While our MT+ complex likely contains a mixture of areas MT and MST in most participants, these studies are potentially informative in explaining the increased BOLD signal during VPI and TC in right MT+ in aged adults. In aged macaques, MT neurons tend to prefer slower speeds, become less speed selective, and become less direction selective compared to neurons measured in MT in young macaques [76,77]. Experiments performed in V1 examining causes of reduced direction and orientation selectivity of neurons in aged macaques suggest that this loss of selectivity could be due to decreased GABA in V1 [78], and a similar mechanism may be at play in MT. Connecting this to our results, it is possible that in aged adults, a greater number of neurons in MT respond to the motion present in our tasks as a result of their reduced speed and direction selectivity, which could also affect activity within area MST. This is one factor that may underlie the stronger response seen in RMT+ in aged adults during both the VPI and TC tasks.

### 4.3. Activity Strength in LMT+, LpVIP, and RpVIP Is Associated with VPI Accuracy

Of the five OF-sensitive regions whose activity during VPI had the strongest inverse relationships with radial motion thresholds, activity in LMT+, LpVIP, and RpVIP during VPI showed positive relationships with VPI performance that were stronger in aged adults than young adults. Activity in LpVIP during TC showed a positive relationship with VPI performance that was similarly stronger in aged adults, as well, suggesting that navigational demands or attention do not strongly affect the relationship between the response to global radial motion in LpVIP and visual path integration accuracy. The lack of significant relationships between VPI accuracy and OF-sensitive region activity strength in young participants is likely due to the near ceiling-level performance on the VPI task in young participants rather than an absence of such a relationship in young participants. It is possible that a more difficult VPI task would reveal similar relationships in young adults and future work should explore this.

In aged adults, stepwise variable selection identified activity in LMT+ and RpVIP during VPI as variables for a multiple regression model predicting VPI performance; the model was significant and activity strength in both regions were significant predictors of VPI performance in the model. The same model built using activity strength in each of these regions during TC was not significant. This suggests that there is some effect of the presence of navigational demands on this relationship, which may be the result of a difference in attention to radial optic flow in each task and how attention modulates the activity within these regions. These findings implicate the response of specific OF-sensitive regions during spatial orienting tasks as a novel factor underlying visual path integration performance in aged adults. This is especially interesting in light of the fact that (1) activity in these regions during the VPI task was inversely associated with sensitivity to radial global motion patterns and (2) the literature strongly implicates MST and VIP in self-motion and heading perception. This suggests that global radial motion sensitivity is associated with how these regions respond to the radial motion experienced when moving through an environment and that stronger activity in these areas while tracking position and orientation within an environment supports better performance in aged adults.

Stronger activity in LMT+, LpVIP, and RpVIP during VPI was associated with more accurate performance on the VPI task in aged adults. There were no significant effects of age on activity strength in these regions, but their average activity levels are potentially informative in gaining a better understanding of whether or not the relationship between activity in these areas and performance in the aged group represents compensatory processes. In other words, is it beneficial for activity in these OF-sensitive regions to appear more similar to that in young participants (who performed at or near ceiling) or different from that in young participants? There was a trend for greater activity in RpVIP during VPI in young adults and the average activity in in LpVIP during VPI was also greater in young adults (though it did not reach trend level) (Figure 5). This suggests that aged adults with activity in LpVIP and RpVIP during VPI that was more similar to that in young adults (on average) was beneficial to their performance. Similarly, there was no significant effect of age on activity in LMT+ during VPI; activity levels were quite similar in both groups with the average activity in aged adults slightly higher than that in young adults (Figure 5). It has been shown that attention modulates activity within the MT+, and specifically within MT and MST, with greater attention to visual motion associated with increased activity and neuronal firing within these regions [79,80,81]. It is possible that in aged adults, attention to global visual motion could be closely associated with VPI performance. The variation in LMT+ activity within the aged group might reflect the level of attention directed towards global visual motion. Examination of the behavior of these regions in tasks in which young adults show greater variance in performance and additional study of these regions during spatial orienting in aged adults is needed to better understand the mechanisms underlying these relationships.

Lastly, exploratory analyses revealed that there was no significant effect of activity strength in canonical spatial navigation regions on VPI accuracy and no significant interaction between age and activity strength in these regions on VPI accuracy (Supplementary Material). Significantly weaker activity strength in the left body of the hippocampus and right tail of the hippocampus during VPI and TC in aged adults relative to young adults (Figure S2) support published findings of weaker hippocampal activity during navigation tasks in aged adults relative to young adults and the effect of age on the systems recruited to perform spatial navigation tasks demonstrated in other fMRI studies in this area [36,38,39,82]. However, the lack of a significant relationship between activity strength in these canonical spatial navigation regions and VPI accuracy is interesting in the context of the significant relationships found between OF-sensitive region activity strength and VPI accuracy in aged adults. The stronger relationship between VPI accuracy and activity strength in OF-sensitive cortical regions relative to activity strength in canonical navigation regions suggests that perceptual processes (i.e., optic flow perception) may be playing a stronger role in aspects of navigation ability in aged adults than was previously thought. It is also possible that these findings are the result of the relatively low learning and memory demands of the VPI task used in this study compared to spatial navigation tasks in other studies in which participants are asked to learn environmental layouts. Future work should examine the relationship between activity strength in OF-sensitive regions and canonical spatial navigation regions in the context of navigation tasks with varying amounts of memory and active path integration required. This would allow for the assessment of how much perceptual and spatial learning and memory systems contribute to spatial navigation tasks that rely more or less on memory.

### 4.4. Limitations

Some limitations should be kept in mind while interpreting these results. One important limitation is the imprecise localization of our OF-sensitive regions due to (1) group-level functional localization and (2) the use of a single coherent motion localizer. In terms of the first point, we defined our OF-sensitive regions at the group level to minimize time in the MRI scanner and participant burden. The coordinates of each OF-sensitive region are based on the average location of peak activity measured during coherent dot motion across all participants. This is more specific than using published coordinates to define regions because our regions are specific to our sample of participants, but it is less specific than functionally identifying each region within each participant. We would expect functional localization at the individual level to lead to stronger relationships between activity strength in our regions and radial motion thresholds as well as performance. In terms of the second point, using additional visual motion and vestibular tasks would also contribute to more precise localization and identification of these OF-sensitive regions. For example, differentiating area MT from MST requires the use of an additional localizer that presents dot motion to each visual hemifield. To more concretely identify the putative homolog of VIP (which is polysensory) in participants, a combination of visual, tactile, auditory, and perhaps vestibular stimulation would be required [15]. For other visual motion areas, confirming their representation of the visual field is important to precise identification and therefore retinotopy would be useful in these cases. In other words, showing participants additional combinations of localizers allows for more precise and confident definitions of these cortical motion areas. In future studies, more precise localization of MT, MST, and/or pVIP and other motion areas in the intraparietal sulcus at the individual level would likely be the most relevant to include based on the literature and the findings reported in the present study.

Performing any task related to perception of self-motion within an environment in an MRI scanner limits full translatability to real-world navigation due to the limited field of view and lack of self-motion-related sensory input beyond vision. In terms of the first point, tasks performed in the MRI scanner are viewed on a screen that limits peripheral visual input, which would otherwise be received when moving through real space. Additional visual input associated with both a larger field of view and stronger optic flow cues has been shown to affect performance on visual navigation tasks, suggesting that peripheral visual input might affect performance on our tasks as well as associated brain activity patterns [26,29]. In terms of the second point, no other sensory input congruent with the self-motion experienced in a navigation task performed in an MRI scanner is available. Typically, vestibular, proprioceptive, and even auditory input would be available if one were walking on these paths in the real world, and given that many of our OF-sensitive regions have been reported to respond to vestibular stimuli, their activity might differ if vestibular input were present [13,14]. The absence of this input is useful in the sense that we were able to isolate the use of visual motion input to keep track of one’s position and orientation in space. However, the unrealistic nature of this set-up and lack of additional sensory input almost guarantees that brain activity differs from what it would look like if our participants performed our tasks in real space.

One last limitation is that we did not collect eye-tracking information as part of this experiment. With this information, we could have examined whether there were differences in the amount of eye movement between young and aged participants and also between the VPI and TC tasks. MT+, pVIP, and CSv all show evidence of differentiating between retinal motion due to eye movement versus environment motion [13,74] and therefore likely receive information related to eye movement. Additionally, MT+ and regions in the dorsal parietal lobe have been shown to respond to eye movements in fMRI studies [58,83]. Thus, eye movement data would be potentially informative in terms of evaluating whether the amount of eye movement was closely associated with the activity in these regions. Though this could be informative, the relationships found between activity in MT+, pVIP, and RCSv during VPI and radial motion thresholds suggest that activity levels in these regions were not overwhelmingly due to eye movements. If activity strength in these regions were overwhelmingly due to eye movements, we would not expect to find significant relationships between activity strength in these regions during VPI and global radial motion thresholds (which were unrelated to eye movements because participants fixated on a central point on the computer screen while these thresholds were measured). Additionally, in the aged group we would not expect activity in these regions to be related to VPI performance if it solely represented the amount of eye movements made during the task. Nevertheless, eye tracking could be informative in future studies examining the relationship between activity in these regions and performance on spatial orienting tasks, especially as a covariate in models examining these relationships.

## 5. Conclusions

The results presented herein show a relatively limited effect of age on the activity strength within OF-sensitive regions while viewing navigationally relevant stimuli and provide novel evidence that activity strength within OF-sensitive cortical regions implicated in self-motion perception is associated with accuracy on a visual path integration task in healthy aged adults. Our results were specific to a subset of OF-sensitive regions implicated in visual self-motion perception whose activity during the VPI task was inversely related to radial global motion thresholds measured outside of the MRI scanner—suggesting that weaker activity in these regions during the VPI task represented a weaker response to the radial global motion characteristic of self-motion present in the task. This weaker response could be the result of attention-related processes or age-related changes in these global visual motion areas and associated circuits. Most fMRI studies on aging and spatial navigation to date have focused on spatial learning and spatial memory. Our findings provide novel support for the hypothesized mechanism by which self-motion perception is a factor contributing to aged adults’ spatial navigation ability [1]. More study should be devoted to the role of sensory processes, particularly global motion perception, in real-time spatial orienting in order to better understand variability in spatial navigation abilities across the lifespan and especially within the aged population. Understanding this variability in aging adults could lead to interventions that might facilitate the interactions between members of the aging population and their environment thereby prolonging independence and enhancing quality of life.

## Figures and Tables

**Table 1:** Optic Flow-Sensitive Regions.

OF-Sensitive Region	MNI152 Coordinates (mm)
LCSv	−12, −20, 42
RCSv	−12, −22, 44
LMT+	−38, −64, 2
RMT+	44, −54, 2
LPIVC	−40, −34, 20
RPIVC	40, −30, 20
RPc	14, −42, 56
LpV6	−12, −78, 34
RpV6	20, −74, 36
LpVIP	−22, −62, 62
RpVIP	22, −60, 62

These 11 OF-sensitive regions were defined at the group level according to their activation strength during coherent motion relative to scrambled motion presented in the optic flow localizer and their proximity to the egomotion-sensitive regions reported in Cardin and Smith [8]. The central coordinates of these regions, which are binary spheres with 5 mm radii, are listed in MNI152 space.

**Table 2:** Participant Demographics and the Effect of Age on VPI and TC Task Performance Measures.

Measure	Young (*n* = 29)	Aged (*n* = 22)
Age (years)	25.2 ± 3.42 (20–34)	70 ± 4.87 (62–80) ***
Sex (F/M)	17 F/12 M	16 F/6 M
Education (years completed)	17.5 ± 2.16 (14–24)	18.4 ± 2.81 (12–24)
Handedness (R/L)	24 R/5 L	22 R/0 L *
MoCA(education-adjusted total score)	n/a	27.8 ± 1.54 (25–30)
VPI Accuracy (% Correct)	97.0% (±5.44) (75%−100%)	80.4% (±22.8) (31.3%−100%) **
TC Accuracy (% Correct)	96.8% (±5.69) (81.3%−100%)	91.8% (±13.3) (50%−100%)
Perceived VPI Accuracy	1.76 (±1.15) (1–6)	2.77 (±1.60) (1–7) **
Perceived VPI Difficulty	2.24 (±1.24) (1–5)	3.96 (±1.50) (2–6) **
Effort Exerted VPI	2.69 (±1.23) (1–5)	4.36 (±1.47) (2–7) **
Perceived TC Accuracy	1.90 (±0.860) (1–4)	2.32 (±1.59) (1–6)
Perceived TC Difficulty	2.00 (±0.802) (1–4)	2.50 (±1.26) (1–5)
Effort Exerted TC	2.28 (±1.07) (1–4)	2.59 (±1.50) (1–5)
Neighborhood Familiarity (% Familiar)	58.6%	50%
Familiarity Not Helpful (% Reporting Not Helpful)	76.5%	90.9%
Updating Strategy (% Endorsing)	82.8%	81.8%

The participant demographics for each group are shown with the mean, standard deviation, and range shown for measures for which they are applicable. The mean age was significantly higher in the aged group, by design. There were also significantly more left-handed participants in the young group compared to the aged group, in which all the participants were right handed. Although sex did not significantly differ between groups, the ratio of females to males in the aged group was higher than that in the young group. Education did not differ between groups. For VPI and TC accuracy, values represent the percent of trials performed correctly. For perceived VPI/TC accuracy, perceived VPI/TC difficulty, and effort exerted on VPI/TC, values closer to 1 represent high accuracy, low difficulty, and minimal effort and values closer to 7 represent low accuracy, high difficulty, and large effort. For familiarity and strategy measures, values represent the percent of participants reporting neighborhood familiarity, reporting that familiarity was *not* helpful, and reporting an updating strategy. * *p* < 0.05, ** *p* < 0.01 *** *p* < 0.0001.

**Table 3:** Models Predicting VPI Accuracy in the Aged Group using LMT+ and RpVIP Activity.

Whole Model Statistics		
	VPI Accuracy, VPI Activity Model	VPI Accuracy, TC Activity Model
**R2**	0.421	0.139
***p* value**	0.0055 **	0.241
**RMSE**	18.2	22.2
**Predictor-Specific Statistics: VPI Accuracy, VPI Activity Model**		
	LMT+ Activity VPI	RpVIP Activity VPI
**Parameter estimate (PE)**	0.78	0.297
**Standard Error**	0.274	0.132
**FDR-corrected *p* value**	0.0205 *	0.0368 *
**PE Confidence Interval (lower 95%, upper 95%)**	0.207, 1.35	0.0201, 0.573
**Predictor-Specific Statistics: VPI Accuracy, TC Activity Model**		
	LMT+ Activity TC	RpVIP Activity TC
**Parameter estimate (PE)**	0.602	0.076
**Standard Error**	0.366	0.173
**FDR-corrected *p* value**	0.233	0.665
**PE Confidence Interval (lower 95%, upper 95%)**	−0.164, 1.37	−0.286, 0.438

Stepwise variable selection was used to select OF-sensitive regions whose activity strength during VPI predicted VPI accuracy in aged adults; LMT+ and RpVIP activity were selected. Activity in LMT+ and RpVIP during VPI were both significant predictors of VPI accuracy and the overall model was significant, as well. Greater activity in each region was associated with better VPI accuracy, as reflected by the positive parameter estimates associated with each variable. A regression model predicting VPI accuracy built using LMT+ and RpVIP activity strength during TC was not significant. The positive parameter estimates of LMT+ and RpVIP activity during TC within that model suggest a relationship with VPI accuracy similar to the relationship between VPI accuracy and activity within these areas measured during VPI. However, the lack of navigational demands during TC appears to weaken their relationship with VPI accuracy. * *p* < 0.05, ** *p* < 0.01.

## Data Availability

The data presented in this study are available on request from Dr. Killiany. The data are not publicly available in order to comply with local institutional review board policies.

## Supplementary Materials

The following are available online at https://www.mdpi.com/2076-3425/11/2/245/s1, Supplementary text, Figure S1: Activity within Control Regions during the VPI and TC Tasks, Figure S2: Effect of Age on Activity Strength within Canonical Navigation Regions.
