# Supplementary material for: Activity Strength within Optic Flow-Sensitive Cortical Regions Is Associated with Visual Path Integration Accuracy in Aged Adults
Source: Brain Sci. 2021 Feb 16;11(2):245. doi: 10.3390/brainsci11020245 (PMC7919670; doi:10.3390/brainsci11020245)
Supplement: Supplementary file 1 [file brainsci-11-00245-s001.pdf]

Supplementary Material

Supplementary Methods

In exploratory analyses, we examined whether there was an effect of age on activity strength within a set of brain regions shown to be important to spatial navigation processes and whether activity strength within these regions during the VPI task was associated with VPI accuracy. These regions will be referred to as canonical navigation regions throughout the supplemental material. The set of canonical navigation regions we chose to examine in these exploratory analyses were the entorhinal cortex, the hippocampus (divided into head, body, and tail), the isthmus cingulate (divided into dorsal and ventral), and the caudate nucleus. To define these regions, recon-all was run on the MNI T1 1mm average template in FreeSurfer v6.0. The left and right entorhinal cortex and caudate nucleus regions of interest (ROIs) were extracted in MNI 152 2mm space.

The hippocampus was divided into head, body, and tail using the method described in [72]. Briefly, the left or right hippocampal ROI (defined in FreeSurfer's recon-all pipeline) was loaded into Freeview and viewed in the sagittal plane. To define the boundary between the head and body of the hippocampus, the editor (first author) first advanced medially to the last slice where the head and the body of the hippocampal ROI were continuous (i.e. connected by ROI voxels). The most narrow point between the head and the body that did not include any portion of the head was selected with the cursor and then viewed in the coronal plane. This was defined as the first slice of the hippocampal body ROI; all coronal slices anterior to the hippocampal ROI were defined as the hippocampal head ROI. To define the boundary between the body and the tail of the hippocampus, the editor advanced posteriorly through coronal sections until the first section in which the fimbria of the fornix was fully evident. This was defined as the first slice of

the hippocampal tail. All coronal slices anterior to this slice and posterior to the head-body hippocampal boundary as defined above were defined as the hippocampal body ROI. All coronal slices including and posterior to this slide were defined as the hippocampal tail ROI. This process was carried out for the left and the right hippocampus separately. Though the entire hippocampus has been shown to be involved in a number of spatial navigation processes, the posterior hippocampus has been frequently associated with spatial navigation ability [73-76].

The left and right isthmus cingulate was dilated by a unit of 1, extracted in MNI 152 2mm space, and then divided into dorsal and ventral regions within FSLview. Voxels in and ventral to the z=44 plane were defined as the ventral isthmus ROI and voxels dorsal to this plane were defined as the dorsal isthmus ROI. This process was carried out for the left and the right isthmus cingulate separately. The ventral isthmus cingulate largely overlaps with the retrosplenial cortex, a region strongly implicated in spatial navigation processes, whereas the dorsal isthmus cingulate largely overlaps with the posterior cingulate, which has been less frequently implicated in spatial navigation processes [76-82].

All ROIs were visually inspected to ensure that they were accurately transformed into volumetric space. The average parameter estimate within each of these regions during the VPI task was extracted as a measure of the average activity strength within that region during visual path integration as described in the Methods section. The same process was carried out to obtain a measure of activity strength during the TC task as a measure of the average activity strength within that region during turn counting.

The effect of age on activity strength within these canonical navigation regions was examined during VPI and during TC using two-tailed t-tests. Regression models were constructed in which VPI accuracy was the dependent variable and activity in a given canonical

navigation region during VPI, age, and an interaction term were the independent variables. These exploratory models were built to assess whether there was a relationship between performance on the VPI task and activity within any canonical navigation regions during VPI and whether age moderated these relationships.

Supplementary Results and Discussion

Effect of Age on Activity Strength in Canonical Navigation Regions

During the VPI task, activity strength in the left hippocampal body (t(49))=2.62, p=0.0118), right hippocampal tail (t(49))=3.04, p=0.0038), left dorsal isthmus cingulate (t(49))=2.96, p=0.0048), and right dorsal isthmus cingulate (t(49))=3.36, p=0.0015) was significantly greater in young adults compared to aged adults. There was greater activity strength during VPI in the left entorhinal cortex in young adults compared to aged adults that reached trend level (t(49))=1.98, p=0.0531). The effect of age on activity strength during TC was highly similar to that found during VPI. During the TC task, activity strength in the left entorhinal cortex (t(49))=2.05, p=0.0455), left hippocampal body (t(49))=2.50, p=0.0157), left dorsal isthmus cingulate (t(49))=2.82, p=0.0069), and right dorsal isthmus cingulate (t(49))=2.73, p=0.0087) was significantly greater in young adults compared to aged adults. Notably, the effect of age on activity strength in the right hippocampal tail was specific to the VPI task. It is possible that the VPI task and TC task recruit different neural populations in the right hippocampal tail and that aging more significantly impacts the population more closely associated with spatial navigation processes (recruited in the VPI task). The right posterior hippocampus has been implicated as being important to spatial navigation ability [73-76]. Aging may negatively affect the ability to recruit this region for spatial navigation processes thereby

shifting aged adults to more heavily rely on OF-sensitive regions for these same processes. This is highly speculative and further study is needed to explore this idea. Supplemental Figure 2 shows the average activity strength within this set of canonical navigation regions during the VPI and TC tasks.

Relationship between VPI Accuracy and Activity Strength in Canonical Navigation Regions during VPI

No significant effects of activity strength within canonical navigation regions measured during VPI on VPI accuracy nor any significant interactions were found. There was a trend-level effect of right ventral isthmus cingulate activity strength measured during VPI on VPI accuracy (β=0.208, t(47)=2.06, p_{FDR}=0.0619) and a trend level interaction between age and right ventral isthmus cingulate activity strength measured during VPI on VPI accuracy (β=-0.193, t(47)=-1.91, p_{FDR}=0.0619) with a stronger relationship between right ventral isthmus cingulate activity strength and VPI accuracy in aged adults (r(20)=0.328, p=0.0684) compared to young adults (r(27)=0.076, p=0.348). No significant effects or interactions were found for right ventral isthmus cingulate activity strength measured during TC. Though it is only a trend-level relationship, these results support the continued importance of the right retrosplenial cortex to spatial navigation processes in aged adults [39].

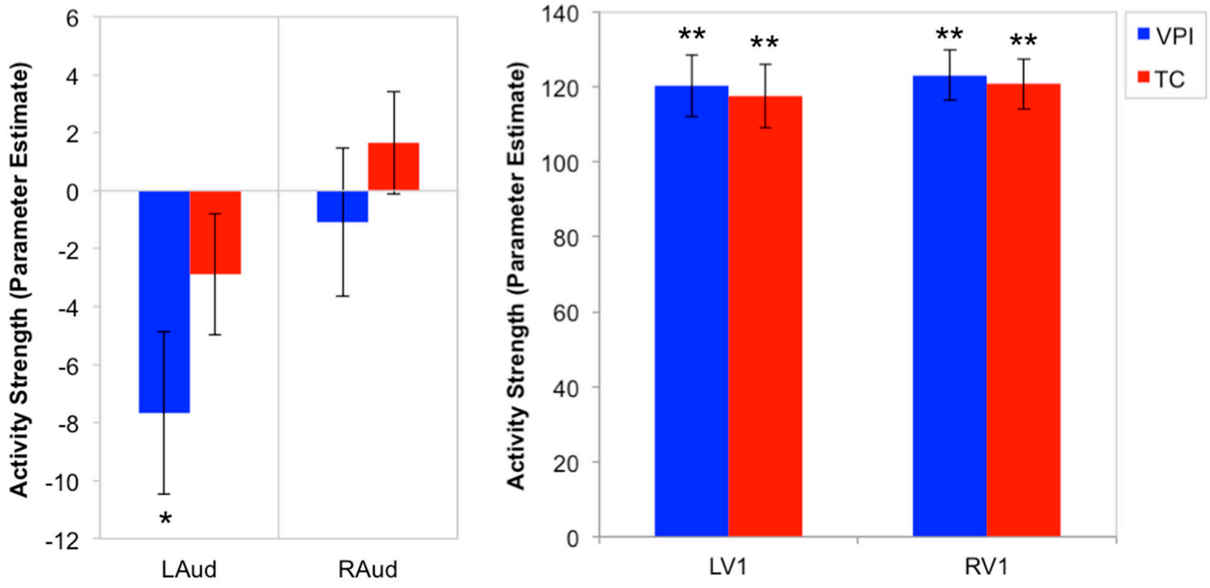

Figure S1: Activity within Control Regions during the VPI and TC Tasks. (left) Average activity within primary auditory cortex was not significantly greater than zero during VPI or TC relative to baseline, as expected. Activity in LAud during VPI was significantly less than zero. (right) Average activity in LV1 and RV1 during VPI and TC relative to baseline was significantly greater than zero, as expected for primary visual cortex. * $p < 0.01$, ** $p < 0.0001$.

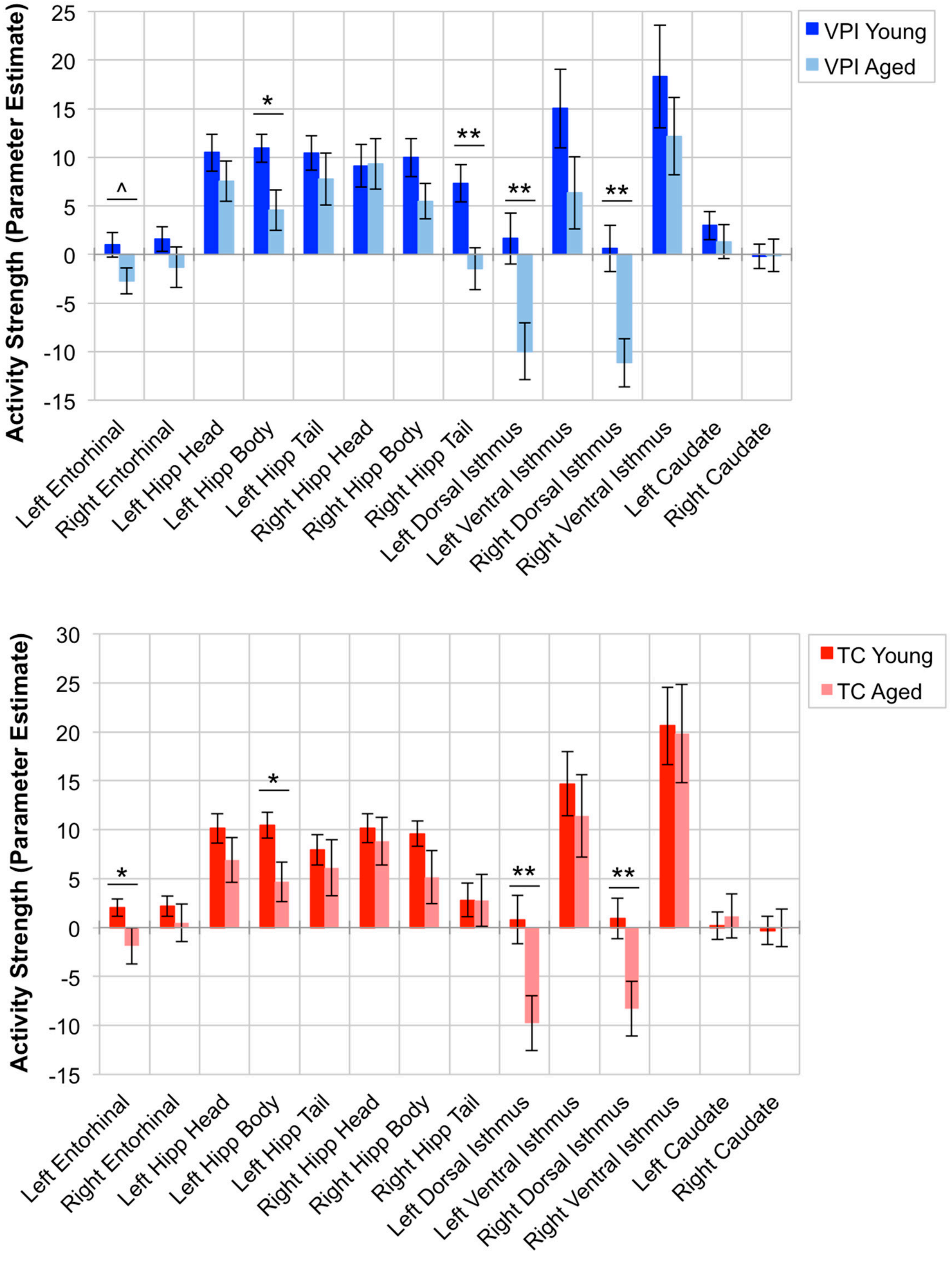

Figure S2: Effect of Age on Activity Strength within Canonical Navigation Regions. In the exploratory analyses discussed in the supplementary material, relationships between activity in

regions that have been shown to be important to spatial navigation processes and VPI performance were assessed and no significant relationships were found. This figure shows the average activity within this set of regions during VPI and TC relative to baseline in the young adults and aged adults. Activity was significantly stronger in the left hippocampal body, right hippocampal tail, left dorsal isthmus cingulate, and right dorsal isthmus cingulate in young adults than in aged adults during the VPI task. Activity strength in the left entorhinal cortex during VPI was stronger in young adults compared to aged adults and reached trend level. Similarly, activity was significantly stronger in the left entorhinal cortex, left hippocampal body, left dorsal isthmus cingulate, and right dorsal isthmus cingulate in young adults than in aged adults during the TC task. ^ $p < 0.06$, * $p < 0.05$, ** $p < 0.01$

Supplemental References

Epstein, R. A. (2008). Parahippocampal and retrosplenial contributions to human spatial navigation. *Trends in Cognitive Sciences, 12*(10), 388-396.

Epstein, R. A., Patai, E. Z., Julian, J. B., & Spiers, H. J. (2017). The cognitive map in humans: Spatial navigation and beyond. *Nature Neuroscience, 20*(11), 1504.

Greene, S. J., & Killiany, R. J. (2012). Hippocampal subregions are differentially affected in the progression to Alzheimer's disease. *The Anatomical Record, 295*(1), 132-140.

Hartley, T., Maguire, E. A., Spiers, H. J., & Burgess, N. (2003). The well-worn route and the path less traveled: Distinct neural bases of route following and wayfinding in humans. *Neuron, 37*(5), 877-888.

Iaria, G., Chen, J., Guariglia, C., Ptito, A., & Petrides, M. (2007). Retrosplenial and hippocampal brain regions in human navigation: Complementary functional contributions to the formation and use of cognitive maps. *European Journal of Neuroscience, 25*(3), 890-899.

Ino, T., Doi, T., Hirose, S., Kimura, T., Ito, J., & Fukuyama, H. (2007). Directional disorientation following left retrosplenial hemorrhage: A case report with fMRI studies. *Cortex, 43*(2), 248-254.

Maguire, E. A., Gadian, D. G., Johnsrude, I. S., Good, C. D., Ashburner, J., Frackowiak, R. S., & Frith, C. D. (2000). Navigation-related structural change in the hippocampi of taxi drivers. *Proceedings of the National Academy of Sciences of the United States of America, 97*(8), 4398-4403. doi:10.1073/pnas.070039597 [doi]

Maguire, E. A., Woollett, K., & Spiers, H. J. (2006). London taxi drivers and bus drivers: A structural MRI and neuropsychological analysis. *Hippocampus, 16*(12), 1091-1101.

Marchette, S. A., Vass, L. K., Ryan, J., & Epstein, R. A. (2014). Anchoring the neural compass: Coding of local spatial reference frames in human medial parietal lobe. *Nature Neuroscience, 17*(11), 1598-1606.

Moffat, S. D., Elkins, W., & Resnick, S. M. (2006). Age differences in the neural systems supporting human allocentric spatial navigation. *Neurobiology of Aging, 27*(7), 965-972.

Takahashi, N., Kawamura, M., Shiota, J., Kasahata, N., & Hirayama, K. (1997). Pure topographic disorientation due to right retrosplenial lesion. *Neurology, 49*(2), 464-469.

Vann, S. D., Aggleton, J. P., & Maguire, E. A. (2009). What does the retrosplenial cortex do? *Nature Reviews Neuroscience, 10*(11), 792-802.

Wolbers, T., & Buchel, C. (2005). Dissociable retrosplenial and hippocampal contributions to successful formation of survey representations. *The Journal of Neuroscience : The Official Journal of the Society for Neuroscience, 25*(13), 3333-3340. doi:25/13/3333 [pii]
